# Late Onset Gestational Diabetes Mellitus: Clinical Implications of Delayed Diagnosis and a Pragmatic Approach to Screening: A Narrative Review

**DOI:** 10.1155/jp/7310599

**Published:** 2026-09-08

**Authors:** Ryosuke Shindo, Shigeru Aoki

**Affiliations:** ^1^ Perinatal Center for Maternity and Neonate, Yokohama City University Medical Center, Yokohama, Japan, yokohama-cu.ac.jp

## Abstract

**Background:**

Gestational diabetes mellitus (GDM) increases maternal and neonatal risks and its reported prevalence has risen with evolving diagnostic criteria and screening strategies. Although screening at 24–28 weeks is widely recommended, optimal testing methods and the value of screening outside that window remain contested. The aim of this narrative review is to critically examine the clinical significance, epidemiology, and pragmatic screening strategies for GDM first identified or retested in late pregnancy (the third trimester) after normal midpregnancy screening.

**Methods:**

This narrative synthesis integrates findings from observational studies, retrospective cohorts, guideline statements, and relevant systematic reviews (including a 2024 meta‐analysis) to summarize current knowledge and highlight gaps.

**Results:**

Reports show that repeat testing can detect new GDM cases. For example, one published retrospective cohort reported an 18.3% incidence among high‐risk women retested in the third trimester. Observational evidence and published reviews indicate associations between late‐diagnosed GDM and higher rates of shoulder dystocia, cesarean delivery, and low 5‐min Apgar scores, though findings for macrosomia, neonatal hypoglycemia, and NICU admission are inconsistent. The short interval between late diagnosis and delivery limits the potential impact of interventions, and diagnosis itself may increase medicalization. Standard IADPSG OGTT thresholds were derived from 24–32‐week cohorts and their applicability in later gestation is uncertain. Pragmatic, selective strategies—targeted retesting based on ultrasound or clinical risk, or screening with random glucose/HbA1c to detect overt hyperglycemia—may reduce unnecessary OGTTs while identifying clinically meaningful cases.

**Conclusions:**

As a narrative review, this article synthesizes current evidence to conclude that late‐onset GDM is clinically relevant but understudied; universal third‐trimester OGTT is not yet supported by robust outcome data. Prospective studies are needed to define optimal screening populations, diagnostic criteria, and management in late pregnancy.

## 1. Introduction

Gestational diabetes mellitus (GDM) poses various risks to both the mother and the fetus, and its prevalence has been increasing worldwide due to recent changes in diagnostic criteria and screening strategies. The optimal timing for GDM screening is internationally agreed upon to be between 24 and 28 weeks of pregnancy [1–3]. However, there is no consensus on the best screening method for GDM, such as whether a one‐step approach or a two‐step approach is better, or whether a 75 g 2‐h oral glucose tolerance test (OGTT) or a 100 g 3‐h OGTT should be used, with recommendations varying by country and region. Additionally, there is disagreement regarding glucose tolerance screening beyond the optimal window (24–28 weeks). Early pregnancy glucose testing is primarily intended to detect overt diabetes rather than GDM. Therefore, screening strategies in early pregnancy are relatively well established. Meanwhile, the role of repeat glucose testing after a normal 24–28‐week screening remains unclear.

The epidemiology, metabolic characteristics, and obstetric implications of these late diagnosed cases have not been fully elucidated. In this narrative review, we critically evaluate the current literature on GDM diagnosed in late pregnancy, with particular emphasis on its clinical implications, limitations of existing screening paradigms, and unresolved questions regarding intervention.

## 2. Epidemiology and Candidates for Retesting in Late Pregnancy

Even when glucose tolerance screening in midpregnancy is normal, repeat testing in case of clinical suspicion may detect new cases of GDM, and some reports have suggested that retesting should be considered in such cases [4–9]. Candidates for retesting in the third trimester include those with relatively high results on the glucose tolerance test in the second trimester [4, 9], a history of GDM [5], or suspected macrosomia [6]. For instance, Boriboonhirunsarn and Sunsaneevithayakul [4] demonstrated that when 823 women who had shown normal results on their initial OGTT or 50 g glucose challenge test (GCT) underwent an additional OGTT, 58 cases (7%) were diagnosed with GDM. Furthermore, Kurtbas et al. [6] reported that among the 194 women who had normal screening results during the second trimester (24–28 weeks), 10 (5.2%) were diagnosed with GDM by repeat screening during the third trimester (30–33 weeks). Compared to women who were not diagnosed with GDM, these women were older and had a higher prevalence of a history of delivering a macrosomic infant (30% vs. 0%).

In a previous retrospective cohort study by Shindo et al. [10], the epidemiology of late‐onset GDM was examined. This study included patients who had a 50 g 1‐h GCT of less than 140 mg/dL between 24 and 26 weeks of gestation but were deemed high‐risk based on pregnancy history (fetal LGA or suspected macrosomia, polyhydramnios, positive urine glucose, etc.) and subsequently underwent a 75 g 2‐h OGTT after 29 weeks of gestation. Of the 238 patients, 49 (18.3%) were newly diagnosed with GDM.

## 3. Glucose Tolerance Screening Methods and Cutoff Values in Late Pregnancy

There is no established method for glucose tolerance screening in late pregnancy. The 75 g 2‐h OGTT proposed by the IADPSG is commonly performed. However, the cutoff values proposed by the IADPSG (FPG ≥ 92 mg/dL, 1 − h ≥ 180 mg/dL, and 2 − h ≥ 153 mg/dL) were established based on the results of the HAPO study [11], which targeted women between 24 and 32 weeks of gestation, and their applicability outside of this gestational period remains uncertain. Physiologically, maternal insulin resistance naturally increases as pregnancy progresses, peaking around 32–34 weeks of gestation. Applying standard midpregnancy diagnostic cutoffs during the late third trimester could therefore lead to overdiagnosis or misclassification of women undergoing expected physiological changes.

In fact, reports have shown that the results of the 75 g 2‐h OGTT vary depending on the pregnancy stage. Iwama et al. [12] observed differences in GDM prevalence when testing populations at less than 14 weeks, 24–28 weeks, and 28–32 weeks, underscoring that glucose dynamics shift significantly over time. Although this analysis did not include groups at gestational ages beyond the scope of the HAPO study (32 weeks or later), it did demonstrate that test values vary depending on gestational age. These findings highlight that extrapolating 24–28‐week criteria to late onset GDM (particularly > 28 weeks) lacks strong physiological validation. Rather than defaulting to IADPSG criteria, robust studies akin to the HAPO study are needed to gather evidence for appropriate, gestation‐specific cutoffs in late pregnancy. Furthermore, the clinical feasibility of conducting a fasting OGTT in the late third trimester is often low due to patient discomfort and logistical challenges.

Additionally, considering the burden of repeated OGTTs, there are reports on screening using HbA1c. A cohort study of approximately 4000 women in China reported that among pregnant women with normal glucose tolerance in midpregnancy, those with HbA1c levels of 5.7% or higher as a screening test in late pregnancy (between 32 and 38 weeks) had a higher incidence of LGA and macrosomia than those with levels below 5.7% [13]. However, the use of HbA1c in late pregnancy is subject to significant physiological limitations. Increased red blood cell turnover and hemodilution from physiological iron deficiency during the third trimester can falsely lower HbA1c values. There is no international consensus on the use of HbA1c for diagnosing GDM in late pregnancy.

## 4. Maternal and Neonatal Outcomes of GDM Diagnosis and Intervention in Late Pregnancy

There are limited reports examining the effects of interventions for GDM diagnosed in late pregnancy. A systematic review and meta‐analysis of 12 observational studies was published in 2024 [14]. This study, which included four prospective and eight retrospective studies published between 1990 and 2022, compared 571 cases of late stage GDM and 3103 cases of women with normal glucose tolerance. All participants had normal results on a 50 g 1‐h GCT or 75 g 2‐h OGTT between 24 and 28 weeks of pregnancy but subsequently underwent further glucose tolerance tests or glucose monitoring. A comparison was conducted between a group diagnosed with GDM based on glucose tolerance tests performed during late pregnancy and a group diagnosed with normal glucose tolerance. The targets for late stage screening were based on ultrasound findings, such as LGA, macrosomia, and polyhydramnios in seven reports [10, 15–19], maternal risk factors in two reports [5, 20], and included low‐risk cases in three reports [21–23]. The meta‐analysis results showed that, compared to the group with normal glucose tolerance on re‐examination in late pregnancy, the group diagnosed with GDM had higher rates of shoulder dystocia (OR 1.57, 95% CI 1.02–2.42), cesarean delivery (OR 1.98, 95% CI 1.51–2.61), and Apgar scores below 7 at 5 min (OR 1.80, 95% CI 1.14–2.86). No differences were observed regarding macrosomia, neonatal hypoglycemia, or neonatal intensive care unit (NICU) admission. This meta‐analysis suggested that, even in patients with GDM diagnosed during late pregnancy, there is a possibility of adverse pregnancy and obstetric outcomes. This diagnosis may be useful for managing delivery and newborn care. However, the diagnosis of GDM may increase the frequency of cesarean deliveries by making it easier for physicians to choose cesarean delivery. Additionally, even if a diagnosis is made at the late stage, the period from the start of the intervention to delivery is limited. This meta‐analysis did not examine differences in outcomes depending on the presence or absence of treatment after diagnosis or the modality of treatment, and concerns remain as to whether intervention is effective even if the diagnosis is made in late pregnancy.

In addition, this meta‐analysis did not include any randomized controlled trials (RCTs), and further prospective studies are needed. Of the 12 studies included in the meta‐analysis, 11 used a two‐step approach involving a 50 g GCT for midpregnancy glucose tolerance screening. One study [5] that used a 75 g OGTT did not examine maternal and fetal outcomes. It is also a topic for future consideration whether the same applies to a population that tested negative using the one‐step approach based on a 75 g OGTT recommended by the IADPSG.

## 5. A Pragmatic Clinical Perspective Based on Accumulated Evidence

The key clinical question is not merely whether late diagnosed GDM can be detected, but whether its detection meaningfully alters maternal and neonatal outcomes. The available evidence [14] suggests that the clinical impact of diagnosing mild glucose intolerance in late pregnancy may be limited, given the short interval between diagnosis and delivery and the modest effectiveness of intervention. Moreover, repeated 75 g OGTTs in the third trimester may increase medicalization without clear perinatal benefit. Nevertheless, the findings of this meta‐analysis should be interpreted as associations rather than evidence of causality. Because all included studies were observational, women diagnosed with late‐onset GDM may have differed from those with normal glucose tolerance in baseline metabolic risk, and residual confounding cannot be excluded. Furthermore, the diagnosis of GDM itself may have influenced obstetric management, including the decision to perform cesarean delivery, thereby contributing to the observed differences in outcomes.

From a pragmatic clinical standpoint, it may therefore be reasonable to prioritize the identification of women with severe hyperglycemia equivalent to overt diabetes mellitus (DM), in whom immediate intervention is likely to influence maternal and neonatal outcomes. In our institution, we have adopted an empirical local protocol that focuses on detecting clinically significant hyperglycemia in late pregnancy using random plasma glucose levels and HbA1c, rather than routinely repeating full OGTTs. Specifically, we target immediate intervention for overt hyperglycemia markers, such as a random plasma glucose level ≥ 200 mg/dL or an HbA1c level ≥ 6.5%. In our experience, implementing this approach has led to a significant reduction in the number of repeated 75 g OGTTs, while cases of overt DM have been identified and treated appropriately. Although further prospective evaluation is warranted, this pragmatic strategy may offer a balanced approach, minimizing unnecessary testing while maintaining vigilance for overt DM in late pregnancy.

## 6. Conclusion

In conclusion, late onset GDM represents a clinically relevant but insufficiently understood entity. Although diagnosis in late pregnancy may identify women at increased risk of certain adverse outcomes, robust evidence demonstrating that intervention at this stage improves prognosis is lacking. Conversely, excessively diagnosing GDM in the third trimester may lead to increased medical costs, heightened anxiety among pregnant women, and concerns among healthcare providers, potentially resulting in unnecessary interventions. A more selective approach focusing on clinically significant hyperglycemia, rather than universal late pregnancy OGTT, may represent a balanced strategy. Future prospective studies are required to clarify the optimal screening population and management paradigm.

## Funding

No funding was received for this manuscript.

## Disclosure

All authors have read and approved the final version of the manuscript. Ryosuke Shindo had full access to all of the data in this study and takes complete responsibility for the integrity of the data and the accuracy of the data analysis.

## Ethics Statement

As this manuscript is a narrative review of existing literature, ethical approval and informed consent were not required.

## Conflicts of Interest

The authors declare no conflicts of interest.

## Data Availability

Data sharing not applicable to this article as no datasets were generated or analyzed during the current study.
